# Antibacterial and Photoactive Niobium Pentoxide-Loaded Chitosan–Gelatin Hydrogels with Favorable Cytocompatibility

**DOI:** 10.3390/ijms27209124

**Published:** 2026-10-15

**Authors:** Muhammad Usman Khalid, Kamilė Kasperavičiūtė, Antanas Straksys, Arunas Stirke, Wanessa De Melo

**Affiliations:** Department of Functional Materials and Electronics, State Research Institute Center for Physical Sciences and Technology (FTMC), 10257 Vilnius, Lithuania; muhammad.usmanbajwa@ftmc.lt (M.U.K.);

**Keywords:** niobium pentoxide, chitosan–gelatin hydrogel, antibacterial activity, singlet oxygen, cytocompatibility, *Staphylococcus aureus*, *Escherichia coli*

## Abstract

The increasing prevalence of multidrug-resistant bacterial infections has created an urgent need for antibacterial biomaterials that combine effective antimicrobial activity with good biocompatibility. In this study, niobium pentoxide (Nb_2_O_5_)-loaded chitosan–gelatin (CG-Nb) hybrid hydrogels containing 1.1% (G1), 1.6% (G2), and 2.0% (G3) (*w*/*w*) Nb_2_O_5_ were evaluated for their antibacterial activity, photo-induced singlet oxygen generation, and cytocompatibility. Antibacterial performance against *Staphylococcus aureus* and *Escherichia coli* was assessed using agar diffusion, direct-contact colony reduction, and scanning electron microscopy, while singlet oxygen generation was investigated using the 9,10-diphenylanthracene (DPA) bleaching assay. Cytocompatibility was evaluated using L929 fibroblasts according to ISO 10993-5. The CG-Nb hydrogels exhibited concentration-dependent antibacterial activity, with G3 showing the highest antibacterial activity among the investigated formulations. G3 produced estimated bacterial reductions of 95.7% against *S. aureus* and 93.3% against *E. coli* in the direct-contact assay, while maintaining 87.3 ± 4.8% L929 fibroblast viability after 24 h. SEM analysis revealed progressive morphological changes in bacterial cells with increasing Nb_2_O_5_ content. The DPA bleaching assay demonstrated that G3 generated photo-induced singlet oxygen under blue-light irradiation. Because the antibacterial assays were performed without blue-light irradiation, the present results do not allow the photo-induced singlet oxygen generation observed in the DPA assay to be directly associated with the antibacterial activity of the hydrogels. Overall, the Nb_2_O_5_-containing chitosan–gelatin hydrogels exhibited enhanced antibacterial activity across the evaluated concentration range while maintaining favorable cytocompatibility under the tested conditions. These findings provide a biological evaluation of the previously developed hydrogel platform and support further investigation of CG-Nb hydrogels for antibacterial biomaterial applications.

## 1. Introduction

Bacterial infections remain a major challenge in biomedical applications, particularly when microorganisms colonize biomaterial surfaces and form persistent biofilms. *Staphylococcus aureus* and *Escherichia coli* are among the clinically relevant bacterial species frequently associated with biomaterial-related infections. The increasing prevalence of antimicrobial resistance further limits the effectiveness of conventional treatments and highlights the need for antibacterial biomaterials that can reduce bacterial colonization while maintaining compatibility with mammalian tissues [1,2]. The growing prevalence of multidrug-resistant strains has intensified the demand for novel antibacterial biomaterials that can circumvent classical resistance mechanisms while remaining safe for mammalian cells [3]. In this context, the design of composite hydrogel systems incorporating bioactive inorganic agents has emerged as a promising strategy for achieving potent, broad-spectrum antibacterial activity alongside favorable cytocompatibility [4].

Chitosan–gelatin (CG) hydrogels are among the most extensively investigated biopolymeric platforms in this field, owing to the complementary properties of their constituent polymers. Chitosan, a natural cationic polysaccharide derived from the deacetylation of chitin, exhibits well-documented intrinsic antibacterial activity through electrostatic interactions with negatively charged bacterial membranes, thereby disrupting membrane integrity and inducing cell death [5]. Gelatin, derived from collagen hydrolysis, provides structural stability, excellent biocompatibility, and cell-adhesive RGD sequences that support mammalian cell attachment and proliferation [6]. The combination of these two biopolymers yields a crosslinked network that synergistically integrates antimicrobial functionality with biomimetic properties conducive to cellular viability [7]. However, the antibacterial efficacy of CG hydrogels alone is often insufficient against highly resistant or biofilm-forming pathogens, necessitating augmentation with additional bioactive components [8].

Metallic nanoparticles, particularly silver (AgNPs) and zinc oxide (ZnO), have been widely incorporated into hydrogel matrices to enhance antibacterial performance. Despite their efficacy, these agents raise notable concerns: AgNPs exhibit significant cytotoxicity toward mammalian cells at bactericidal concentrations, and both silver and zinc oxide nanoparticles have been associated with the induction of microbial resistance mechanisms and potential environmental hazards arising from their accumulation and ion release [9,10]. These limitations have driven the search for alternative bioactive inorganic agents with more favorable biological profiles.

Niobium pentoxide (Nb_2_O_5_) nanoparticles have recently attracted growing interest as a biocompatible inorganic additive with emerging antimicrobial potential. Niobium is a well-established biocompatible element, long utilized in titanium-niobium alloys for orthopedic and dental implants owing to its corrosion resistance, low cytotoxicity, and favorable tissue integration [11]. Nb_2_O_5_ has also been investigated for its favorable biocompatibility and relatively low cytotoxicity, supporting its consideration for incorporation into antibacterial biomaterials [12].

Compared with other commonly investigated metal oxide nanoparticles such as Ag, ZnO, CuO, and TiO_2_, niobium pentoxide has attracted attention because of its chemical stability, favorable biocompatibility, relatively low cytotoxicity, and reported photoactive properties. These characteristics support further investigation of Nb_2_O_5_ as a component of antibacterial biomaterials where chemical stability and biocompatibility are important considerations. Its potential to provide antibacterial functionality while maintaining compatibility with mammalian cells supports further investigation of Nb_2_O_5_-containing hydrogel systems.

Although our previous studies established the physicochemical characteristics of Nb_2_O_5_-loaded chitosan–gelatin hydrogels, their biological performance has not yet been systematically evaluated. The present study therefore investigated the antibacterial activity of these hydrogels against representative Gram-positive and Gram-negative bacteria, evaluated photo-induced singlet oxygen generation under blue-light irradiation, and assessed their in vitro cytocompatibility according to ISO 10993-5. The findings provide a biological evaluation of this previously developed hydrogel platform and contribute to understanding its potential for antibacterial biomaterial applications.

## 2. Results and Discussions

### 2.1. Antibacterial Activity

The antibacterial efficacy of chitosan–gelatin (CG) hydrogels incorporating different concentrations of niobium pentoxide (Nb_2_O_5_) nanoparticles was evaluated against *Staphylococcus aureus* and *Escherichia coli* using the agar well diffusion method. Zone of inhibition (ZOI) diameters, including those for the 10 mm hydrogel disc, were measured after 18–24 h of incubation at 37 °C. All experiments were performed in triplicate, and results are expressed as mean ± standard deviation (SD) in Table 1. Control plates containing only bacterial lawns (without hydrogel discs) showed no zones of inhibition, providing a baseline for comparison with the hydrogel formulations.

Statistical analysis was performed as described in Section 3.5. Statistical analysis revealed significant differences among the formulations. One-way ANOVA showed significant variation in antibacterial activity against both *S. aureus* (F(3,8) = 156.3, *p* < 0.001) and *E. coli* (F(3,8) = 142.7, *p* < 0.001). Post hoc comparisons using Tukey’s HSD test confirmed that: (1) All Nb_2_O_5_-containing formulations (G1, G2, G3) exhibited significantly higher antibacterial activity compared to the CG control (*p* < 0.05 for G1, *p* < 0.01 for G2, *p* < 0.001 for G3; Tukey’s post hoc test). (2) The G3 formulation showed the highest statistical significance (*p* < 0.001) compared to all other groups. These statistical findings demonstrate significant differences in antibacterial activity among the tested formulations, with activity increasing across the evaluated Nb_2_O_5_ concentration range [13].

The plain CG hydrogel (without Nb_2_O_5_ nanoparticles) exhibited moderate antibacterial activity, producing inhibition zones of 12.8 ± 0.6 mm against *S. aureus* and 11.4 ± 0.5 mm against *E. coli*. This inherent antibacterial activity has been associated with the polycationic nature of chitosan, in which protonated amino groups (NH_3_^+^) can interact electrostatically with negatively charged bacterial cell surfaces [14,15]. Slightly higher susceptibility of *S. aureus* compared to *E. coli* is consistent with the established literature. This can be explained by structural differences in their cell walls: *S. aureus* lacks the outer lipopolysaccharide membrane present in Gram-negative bacteria, which serves as an additional permeability barrier [16].

**Figure 1:**
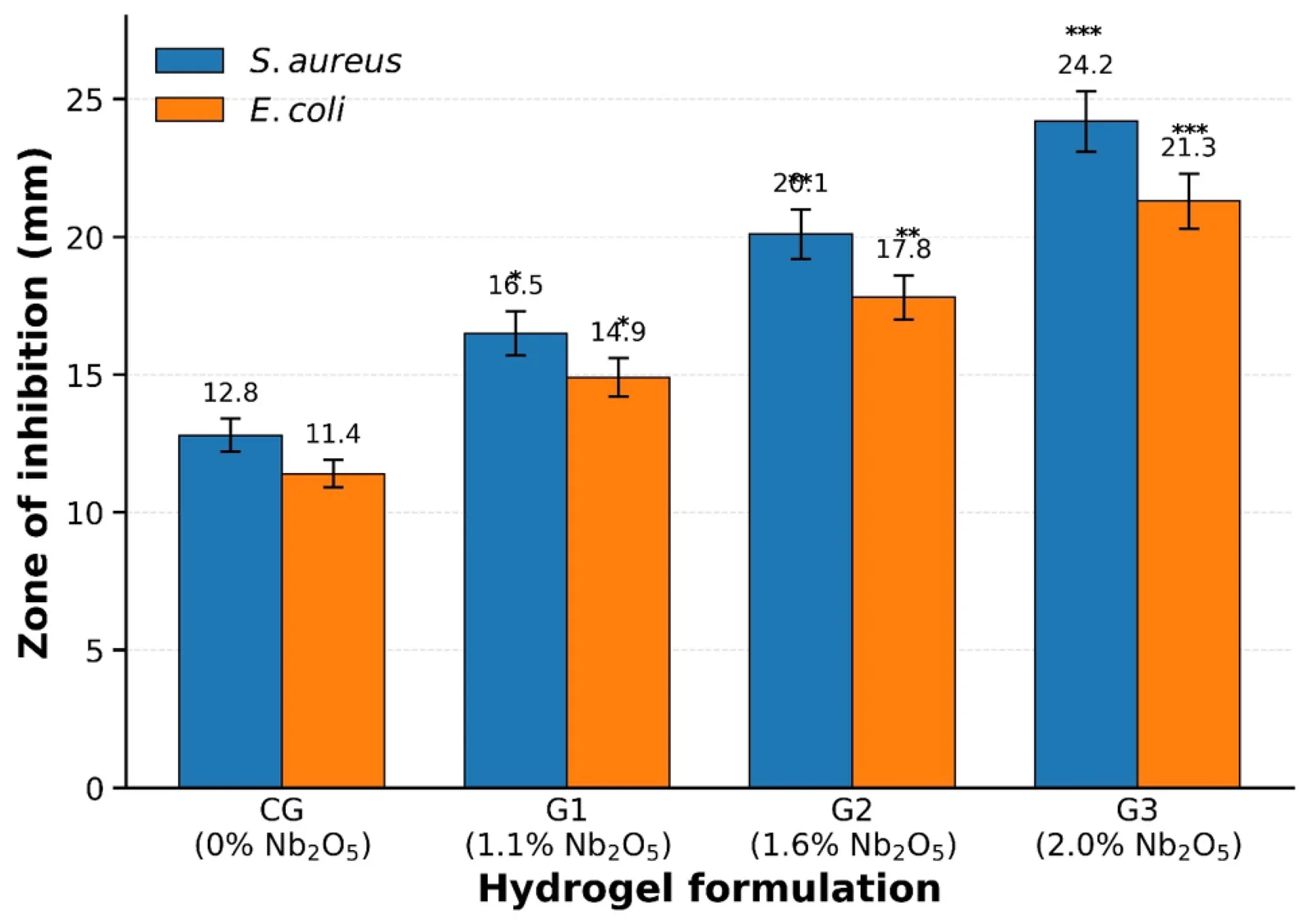
Zone of inhibition (ZOI) diameters of CG and Nb_2_O_5_-loaded hydrogels (G1–G3) against *S. aureus* and *E. coli*. Data represent the mean ± SD (*n* = 3). Asterisks indicate statistically significant differences from the CG control: * = *p* < 0.05, ** = *p* < 0.01, and *** = *p* < 0.001 (one-way ANOVA with Tukey’s post hoc test).

Incorporation of Nb_2_O_5_ nanoparticles into the CG matrix significantly enhanced the antibacterial activity in a concentration-dependent manner (Table 1). The G1 formulation (1.1% *w*/*w* Nb_2_O_5_) produced ZOIs of 16.5 ± 0.8 mm against *S. aureus* and 14.9 ± 0.7 mm against *E. coli*, representing approximately 29% and 31% increases over the CG control, respectively. As the Nb_2_O_5_ content increased, the antibacterial efficacy progressively improved, with G2 (1.6% *w*/*w*) showing ZOIs of 20.1 ± 0.9 mm against *S. aureus* and 17.8 ± 0.8 mm against *E. coli* (57% and 56% increases). Notably, the G3 formulation (2.0% *w*/*w* Nb_2_O_5_) demonstrated the highest antibacterial activity, with maximum ZOIs of 24.2 ± 1.1 mm against *S. aureus* and 21.3 ± 1.0 mm against *E. coli*, corresponding to approximately 89% and 87% increases compared to the plain CG hydrogel, as shown in Figure 1.

Because the antibacterial assays were conducted without external light irradiation, the observed antibacterial activity cannot be directly attributed to photo-induced singlet oxygen generation. The antibacterial activity under these conditions may be primarily associated with the intrinsic antibacterial properties of chitosan, in which protonated amino groups interact electrostatically with negatively charged bacterial cell surfaces and may affect bacterial membrane integrity. Nb_2_O_5_ incorporation may further contribute to the observed antibacterial response through interactions between the composite hydrogel and bacterial cells; however, its specific mechanism under dark conditions was not directly investigated in this study. Therefore, the observed antibacterial activity is considered to result from the combined contributions of the chitosan matrix and Nb_2_O_5_ incorporation. Further experiments using matched light and dark antibacterial conditions would be required to directly assess the contribution of photoinduced activity to antibacterial performance.

The progressive increase in ZOI with increasing Nb_2_O_5_ content indicates enhanced antibacterial activity within the tested concentration range of 1.1–2.0% (*w*/*w*). Concentrations above 2.0% (*w*/*w*) were not evaluated because preliminary formulation trials resulted in nanoparticle aggregation and inhomogeneous hydrogel formation [17].

The G3 formulation (2.0% *w*/*w*) showed the highest activity among the evaluated compositions. However, concentrations above 2.0% (*w*/*w*) were not evaluated because preliminary formulation trials resulted in nanoparticle aggregation and inhomogeneous hydrogel formation. Therefore, G3 should be considered the highest-performing formulation among those successfully evaluated rather than an optimal or ideal composition [18].

This differential susceptibility may be related to structural differences between the two bacterial cell envelopes. The outer membrane of Gram-negative bacteria contains lipopolysaccharides and can provide an additional permeability barrier to antimicrobial agents [19]. Differences in membrane-associated transport mechanisms may also influence bacterial susceptibility [20].

### 2.2. Direct Contact Colony Reduction Assay

The antibacterial activity of the chitosan–gelatin (CG) hydrogel and its Nb_2_O_5_ nanocomposite formulations (G1, G2, G3) was quantitatively evaluated using a direct contact colony reduction assay against *Staphylococcus aureus* and *Escherichia coli.* This method provides a quantitative assessment of bactericidal activity by directly exposing the hydrogel to bacterial cells in a liquid medium for 24 h. The percentage reduction in colony-forming units (CFU) was calculated relative to untreated control wells. All experiments were performed in triplicate with three independent hydrogel discs per formulation (*n* = 3). Results are expressed as mean ± standard deviation (SD) in Table 1. The CG control (without Nb_2_O_5_) showed colony counts exceeding 300 CFU for both strains, consistent with the moderate antibacterial activity observed in the agar diffusion assay (Section 3.1). Accurate reduction percentages could not be calculated for CG because the colony counts exceeded the countable range. Importantly, the absence of a quantifiable CFU reduction in the direct-contact assay does not indicate that the CG hydrogel lacked antibacterial activity. The agar diffusion assay demonstrated measurable inhibition zones of 12.8 ± 0.6 mm against *S. aureus* and 11.4 ± 0.5 mm against *E. coli*. This intrinsic antibacterial activity is consistent with the well-established interaction of protonated chitosan amino groups with negatively charged bacterial surfaces. The different observations between the two assays may be related to their different experimental endpoints: the agar diffusion assay evaluates inhibition-zone formation around the hydrogel, whereas the direct-contact assay quantifies recoverable viable bacteria after 24 h of direct exposure. Thus, moderate antibacterial activity of the CG hydrogel can be detected by agar diffusion, while the remaining viable bacterial population in the direct-contact assay remains above the quantifiable range. The Nb_2_O_5_-containing formulations (G1, G2, and G3) showed progressively lower colony counts with increasing Nb_2_O_5_ content for both bacterial strains. Accurate reduction percentages could not be calculated for CG because colony counts exceeded the countable range.

The apparent difference between the two assays may also reflect the physicochemical characteristics of chitosan and the specific conditions used for the antibacterial evaluation. The antibacterial activity of chitosan is influenced by factors such as the degree of deacetylation, molecular weight, protonation of amino groups, polymer concentration, and environmental pH. Under the present conditions, the intrinsic antibacterial activity of the chitosan-containing CG matrix was sufficient to produce measurable inhibition in the agar diffusion assay but did not result in a quantifiable reduction in recoverable CFU in the direct-contact assay. Therefore, the absence of a quantifiable CFU reduction for CG should not be interpreted as an absence of intrinsic chitosan-mediated antibacterial activity.

The colony counts progressively decreased with increasing Nb_2_O_5_ content, consistent with the representative agar plate images provided in the Supplementary Materials (Figures S1 and S2). For *S. aureus*, the colony counts decreased from 53 CFU for G1 to 37 CFU for G2 and further to 13 CFU for G3. Similarly, for *E. coli*, the colony counts decreased from 56 CFU for G1 to 32 CFU for G2 and to 20 CFU for G3. The G1 formulation (1.1% *w*/*w* Nb_2_O_5_) showed an estimated 82.3% reduction against *S. aureus* and 81.3% against *E. coli*. As the Nb_2_O_5_ content increased to 1.6% *w*/*w*, G2 showed lower colony counts, corresponding to estimated reductions of 87.7% against *S. aureus* and 89.3% against *E. coli*. The G3 formulation (2.0% *w*/*w* Nb_2_O_5_) showed the lowest colony counts among the evaluated formulations, corresponding to estimated reductions of 95.7% against *S. aureus* and 93.3% against *E. coli*.

Statistical analysis revealed significant differences among the formulations. One-way ANOVA, including all four groups (CG, G1, G2, G3), showed significant variation in bactericidal activity against both *S. aureus* (F(3,8) = 52.3, *p* < 0.001) and *E. coli* (F(3,8) = 41.7, *p* < 0.001). Tukey’s post hoc test confirmed significant differences between CG and all Nb_2_O_5_-containing groups (*p* < 0.05 for G1, *p* < 0.01 for G2 and G3) and among G1, G2, and G3 (*p* < 0.05 for all pairwise comparisons). These statistical findings demonstrate significant differences in colony counts among the tested formulations, with lower colony counts observed across the evaluated Nb_2_O_5_ concentration range [21]. The coefficient of variation (CV) ranged from 1.0% to 3.8% across all measurements, indicating relatively low variability among replicate measurements [20].

The G3 formulation showed slightly higher activity against *S. aureus* (95.7% reduction) compared to *E. coli* (93.3% reduction). The approximately 2.4% difference may be related to structural differences between the bacterial cell envelopes [22]. *E. coli* possesses an outer membrane composed of lipopolysaccharides (LPS) that acts as an effective permeability barrier, which can act as an additional permeability barrier to antimicrobial agents [23].

The observed differences in antibacterial response should therefore be interpreted as strain-dependent variations rather than evidence of a distinct antibacterial mechanism. The higher susceptibility observed for *S. aureus* in the present study may reflect differences in the interaction of the hydrogel components with the respective bacterial cell envelopes. However, the current experiments were not designed to distinguish the contributions of specific physicochemical or cellular mechanisms. Further mechanistic studies would be required to determine how Nb_2_O_5_ incorporation influences bacterial responses at the cellular level.

Overall, the colony reduction assay showed that the Nb_2_O_5_-containing hydrogels produced lower colony counts than the CG control, with progressively lower counts observed from G1 to G3 for both bacterial strains. G3 showed the lowest colony counts among the evaluated formulations.

### 2.3. Singlet Oxygen Generation (DPA Bleaching Assay)

**Figure 2:**
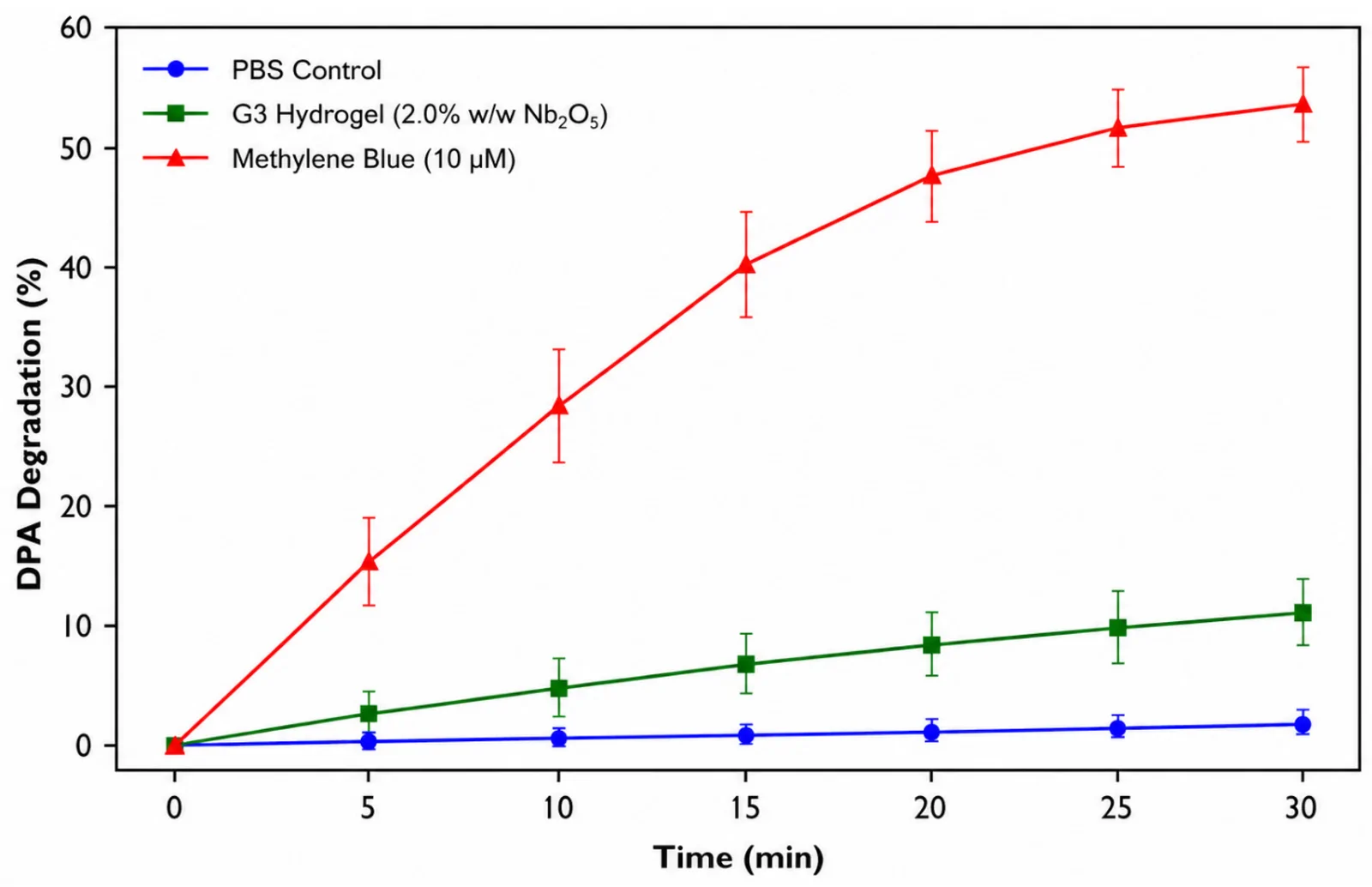
Time-dependent bleaching of 9,10-diphenylanthracene (DPA) under blue light irradiation (450 ± 10 nm, 50 mW/cm^2^) over 30 min. The graph shows DPA bleaching (%) for the G3 hydrogel (2.0% *w*/*w* Nb_2_O_5_), methylene blue (positive control, 10 µM), and PBS (negative control). Data represent mean ± SD (*n* = 3).

The capacity of the G3 formulation (2.0% *w*/*w* Nb_2_O_5_) to generate singlet oxygen (^1^O_2_) under blue light irradiation was assessed using the 9,10-diphenylanthracene (DPA) bleaching assay, which exploits the selective reaction of ^1^O_2_ with DPA to form a non-fluorescent endoperoxide, quantifiable as a decrease in absorbance at 374 nm. The time-dependent DPA bleaching profiles for G3, methylene blue (positive control), and PBS (negative control) under irradiation (450 ± 10 nm, 50 mW/cm^2^) are presented in Figure 2.

The G3 hydrogel was selected for the DPA experiment because it exhibited the highest antibacterial activity among all investigated formulations. The objective of this experiment was to determine whether the most active hydrogel possessed the ability to generate photo-induced singlet oxygen rather than to establish a quantitative relationship between Nb_2_O_5_ concentration and singlet oxygen production. The PBS negative control exhibited negligible DPA bleaching throughout the experiment, reaching only 1.3 ± 0.4% at 30 min, indicating minimal bleaching under the irradiation conditions employed [24]. The G3 hydrogel demonstrated time-dependent, progressive DPA bleaching under irradiation, increasing from 0% at baseline to 11.2 ± 2.2% at 30 min, with a consistent linear trend across all measured time points. These results demonstrate that the G3 hydrogel can generate photo-induced singlet oxygen under the blue-light irradiation conditions used in the DPA assay.

Since the DPA bleaching assay is selective for singlet oxygen (^1^O_2_), the present results specifically demonstrate photo-induced singlet oxygen generation under the experimental conditions employed [25]. The G3 hydrogel also exhibited substantial reductions in colony counts, with estimated reductions of 95.7% against *S. aureus* and 93.3% against *E. coli*; however, because the antibacterial assays were conducted without external irradiation, these results cannot be directly attributed to the photo-induced singlet oxygen detected in the DPA assay.

Taken together, the DPA results demonstrate that the G3 hydrogel can generate photo-induced singlet oxygen under blue-light irradiation. However, because the antibacterial assays were conducted without external irradiation, the present data do not establish that photo-induced singlet oxygen contributes directly to the antibacterial activity observed in those assays. The antibacterial effects may reflect the combined contributions of the intrinsic antibacterial properties of chitosan and Nb_2_O_5_ incorporation. Further experiments using matched light and dark antibacterial conditions would be required to determine the contribution of photo-induced processes to antibacterial activity.

### 2.4. In Vitro Cytotoxicity (XTT Assay)

The cytocompatibility of the CG-Nb hydrogel series was evaluated against L929 mouse fibroblast cells using the XTT assay under direct contact conditions, in accordance with ISO 10993-5 guidelines [26]. The mean absorbance values and corresponding cell viability percentages for all formulations are presented in Table 2. According to ISO 10993-5, materials are classified as non-cytotoxic when cell survival exceeds 70% relative to the untreated control after 24 h of exposure. All formulations tested in this study remained well above this threshold [27].

The untreated control group had a mean absorbance of 3.425 ± 0.235 OD, corresponding to 100% baseline viability. The G1 formulation (1.1% *w*/*w* Nb_2_O_5_) produced a mean absorbance of 3.376 ± 0.120 OD, corresponding to a cell viability of 98.6 ± 3.5%, effectively indistinguishable from the control. G2 (1.6% *w*/*w* Nb_2_O_5_) yielded 94.2 ± 4.1% viability, and G3 (2.0% *w*/*w* Nb_2_O_5_) produced the lowest recorded viability of 87.3 ± 4.8%. Importantly, even at the highest Nb_2_O_5_ concentration tested, cell viability remained above the ISO 10993-5 cytotoxicity threshold, indicating no evidence of cytotoxicity under the 24 h assay conditions.

A modest concentration-dependent decline in viability was observed across G1–G3, with a difference of approximately 11% between the lowest and highest Nb_2_O_5_ loadings. The decrease in viability with increasing Nb_2_O_5_ content indicates that nanoparticle loading may influence fibroblast metabolic activity under the conditions tested. However, the present study evaluated cytocompatibility only after 24 h of exposure, and the XTT assay measures metabolic activity rather than specific cellular mechanisms of toxicity. Therefore, longer-term effects and the mechanisms underlying the observed concentration-dependent decrease cannot be established from the current data. Nevertheless, all formulations remained above the ISO 10993-5 threshold under the tested conditions.

The relatively high antibacterial activity together with the maintained fibroblast viability may be related to the different interactions of the hydrogel components with bacterial and mammalian cells. The cationic chitosan component can interact with negatively charged bacterial surfaces, contributing to antibacterial activity, while the gelatin component provides a biocompatible matrix that may support mammalian cell compatibility. In addition, Nb_2_O_5_ incorporation enhanced the antibacterial activity, while the highest loading tested (G3) maintained 87.3 ± 4.8% fibroblast viability after 24 h. However, the present study did not directly investigate nanoparticle release, cellular uptake, membrane integrity, or other mechanisms that could explain this differential biological response. Therefore, the observed antibacterial activity and cytocompatibility should be considered under the specific experimental conditions used, and the underlying mechanism cannot be established from the current data [28,29].

The G3 formulation, which showed the highest antibacterial activity among the evaluated formulations, maintained 87.3 ± 4.8% fibroblast viability after 24 h of exposure. This finding indicates that G3 remained above the ISO 10993-5 cytotoxicity threshold under the conditions tested. However, because the cytocompatibility assessment was limited to a 24 h XTT assay and was performed without external irradiation, these results should be considered an initial assessment rather than evidence of long-term biocompatibility or a therapeutic index. Further studies involving longer exposure periods and matched light/dark conditions would be required to establish the broader biological performance of the hydrogel system.

Pristine Nb_2_O_5_ was not evaluated as an independent experimental group for antimicrobial activity or cytotoxicity in the present study. The experimental design focused on Nb_2_O_5_-loaded chitosan–gelatin hydrogels, with the unloaded chitosan–gelatin hydrogel (CG) serving as the matrix control. Therefore, the present experimental design does not allow the biological contribution of pristine Nb_2_O_5_ to be completely distinguished from its effects after incorporation into the hydrogel matrix. Direct evaluation of pristine Nb_2_O_5_ under the same experimental conditions would provide a more complete understanding of the individual and combined contributions of the nanoparticles and polymeric matrix and is therefore identified as a limitation and a subject for future investigation.

### 2.5. Scanning Electron Microscopy Analysis of Antibacterial Activity

**Figure 3:**
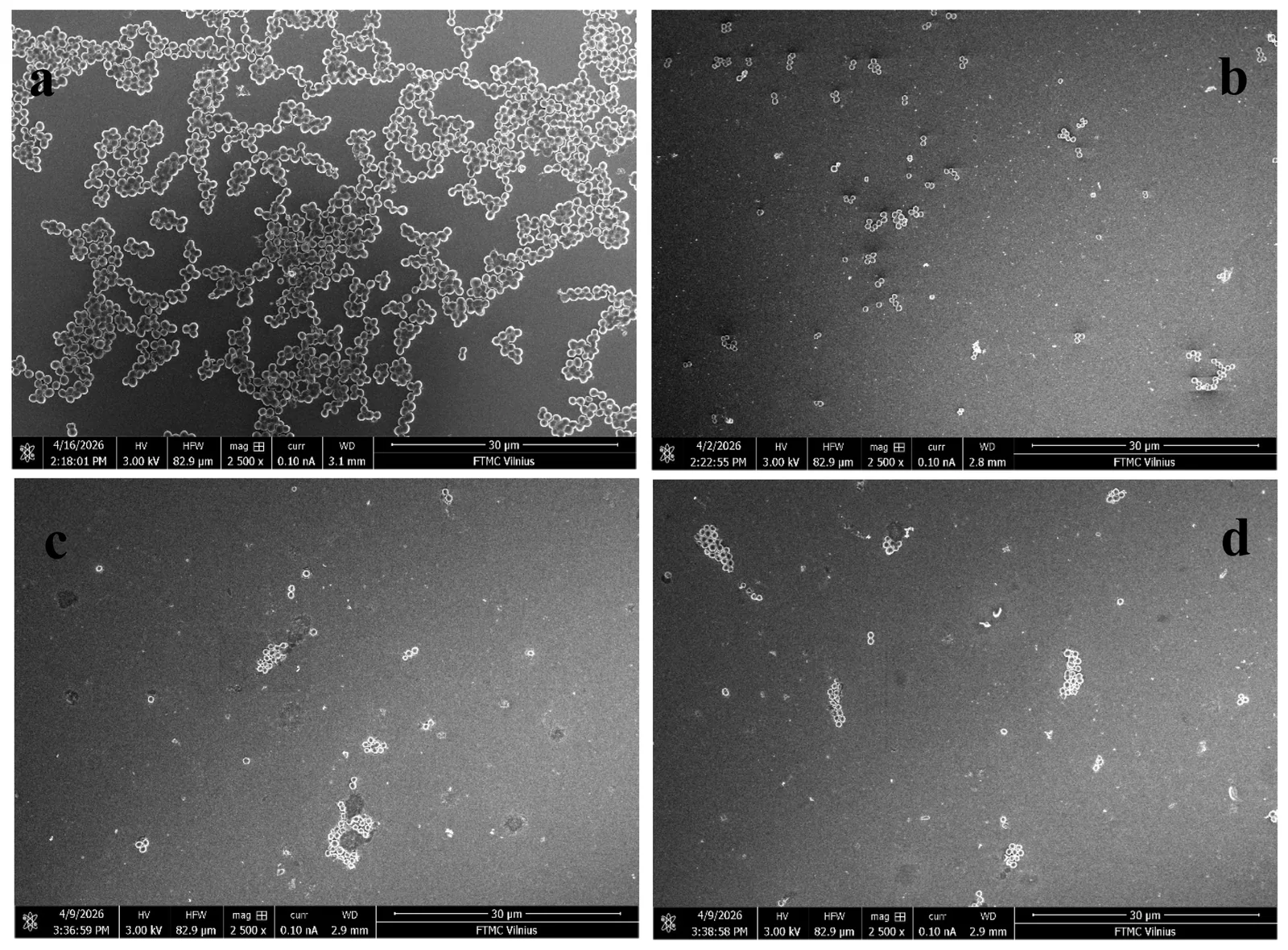
SEM images of *S. aureus* after 24 h contact with CG-Nb hydrogels. (**a**) Control. (**b**) G1 (1.1% Nb_2_O_5_). (**c**) G2 (1.6% Nb_2_O_5_). (**d**) G3 (2.0% Nb_2_O_5_), Scale bar = 30 µm.

**Figure 4:**
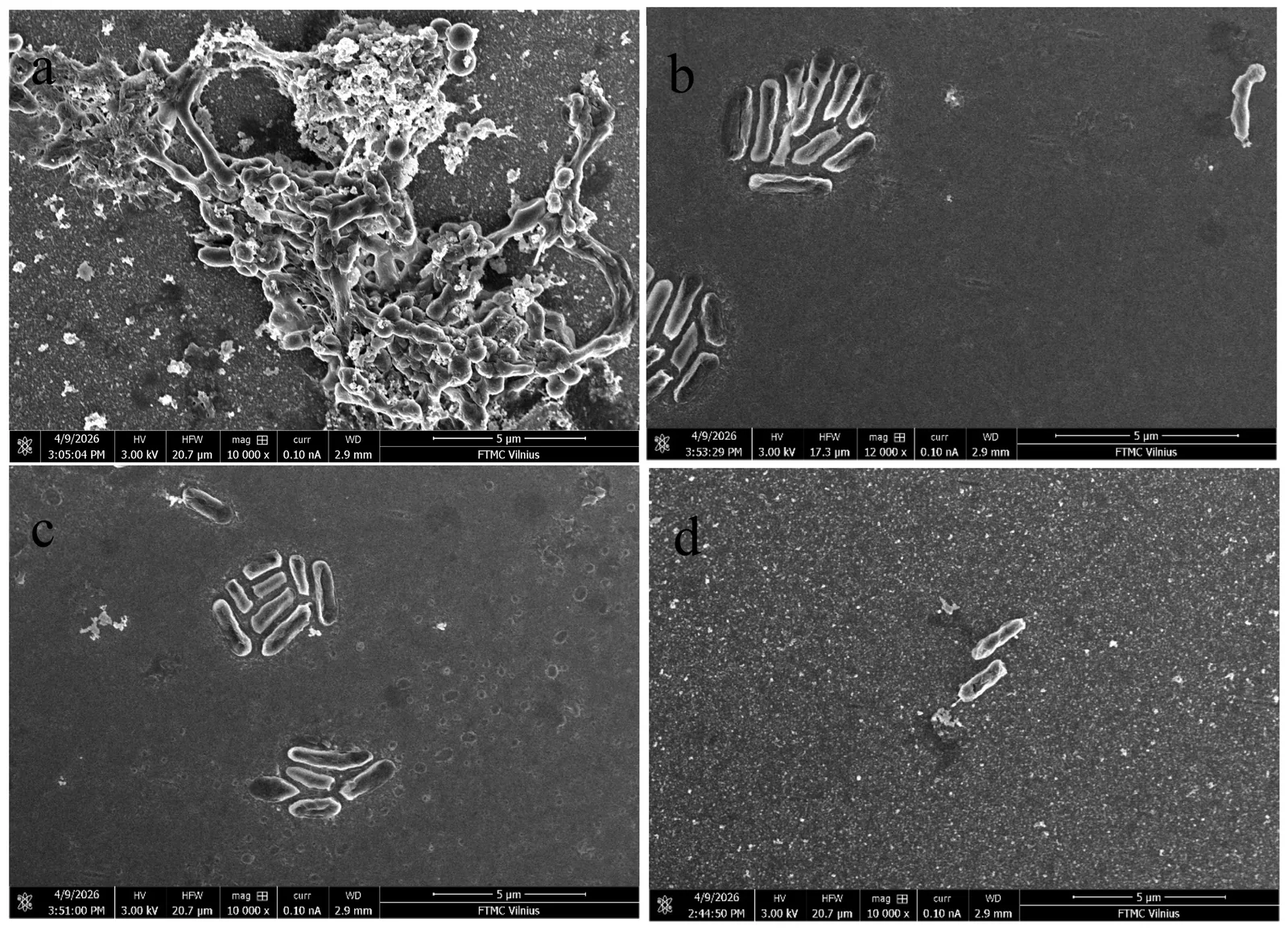
SEM images of E. coli after 24 h contact with CG-Nb hydrogels. (**a**) Control. (**b**) G1 (1.1% Nb_2_O_5_). (**c**) G2 (1.6% Nb_2_O_5_). (**d**) G3 (2.0% Nb_2_O_5_). Scale bar = 5 µm.

Scanning electron microscopy (SEM) was employed to provide morphological changes in the antibacterial activity of CG-Nb hydrogels against both *Staphylococcus aureus* (ATCC 25923) and *Escherichia coli* (ATCC 25922) and to provide qualitative morphological support for the quantitative results obtained from the colony reduction assay. Representative micrographs of untreated control and G1-, G2-, and G3-treated bacterial populations for each strain are presented in Figure 3 and Figure 4, respectively.

The untreated control (Figure 3a) displayed the characteristic morphological features of healthy *S. aureus*: densely packed, spherical cocci of uniform size arranged in large grape-like clusters, with smooth, intact, well-defined cell surfaces and no evidence of membrane deformation. *Staphylococcus aureus* is a bacterium approximately 0.8 µm in diameter, characteristically arranged in grape-like clusters under electron microscopy, a pattern clear in the control micrograph [30]. The extensive clustering and high bacterial density across the imaged surface confirmed the establishment of a robust bacterial population in the absence of any antimicrobial treatment, providing a reliable morphological baseline for comparison with treated groups [31].

Exposure to the G1 formulation (1.1% *w*/*w* Nb_2_O_5_, Figure 3b) resulted in a pronounced reduction in bacterial density relative to the control. The dense clusters characteristic of untreated *S. aureus* were replaced by sparse, isolated microcolonies and individual cocci distributed across a largely unoccupied surface. While the remaining cells retained a broadly intact spherical morphology at this concentration, the reduced population density was consistent with the estimated 82.3% bacterial reduction observed in the direct-contact assay [32].

In micrographs of G2-treated bacteria (1.6% *w*/*w* Nb_2_O_5_, Figure 3c), the reduction in bacterial population was further pronounced, with only scattered individual cells and occasional small clusters remaining visible. Importantly, a subset of the residual cells displayed irregular surface contours and less sharply defined outlines than control cocci, suggesting morphological alterations of the bacterial cell envelope at this intermediate Nb_2_O_5_ concentration.

The most pronounced morphological effects were observed in the G3-treated group (2.0% *w*/*w* Nb_2_O_5_, Figure 3d). Bacterial density was reduced to a minimal level, with only a small number of cells detectable across the entire imaged field. The residual bacteria exhibited altered morphology, including irregular outlines and surface features. These morphological changes are consistent with cellular damage following exposure to the CG-Nb hydrogels; however, SEM observations alone cannot establish membrane leakage, cytoplasmic loss, or a specific mechanism of cell death. The observed structural changes were consistent with the antibacterial activity measured in the colony reduction assay [33,34]. The lower-magnification images in Figure 3 were selected to provide a wider field of view and allow visualization of the overall bacterial population and the pronounced reduction in bacterial density following hydrogel treatment. Higher-magnification imaging was used where detailed individual-cell morphology was required.

The untreated control (Figure 4a) displayed the characteristic morphological features of healthy *E. coli*: densely packed, uniformly sized, rod-shaped bacilli distributed across the substrate surface, with smooth, intact, well-defined cell surfaces and no evidence of membrane deformation. *Escherichia coli* is a bacterium approximately 1–3 µm in length and 0.25–1.0 µm in diameter, characteristically appearing as discrete rods or short chains under electron microscopy, a pattern clearly visible in the control micrograph [35]. The high bacterial density and morphological uniformity across the imaged surface confirmed the establishment of a robust bacterial population in the absence of any antimicrobial treatment, providing a reliable morphological baseline for comparison with treated groups [36].

Exposure to the G1 formulation (1.1% *w*/*w* Nb_2_O_5_, Figure 4b) resulted in a pronounced and visually striking reduction in bacterial density relative to the control. The dense rod-shaped population characteristic of untreated *E. coli* was replaced by sparse, isolated microcolonies and small clusters of individual bacilli distributed across a largely unoccupied surface. While the remaining cells retained a broadly intact rod-shaped morphology at this concentration, the reduced population density was consistent with the estimated 81.3% bacterial reduction observed in the direct-contact assay [37].

In micrographs of G2-treated bacteria (1.6% *w*/*w* Nb_2_O_5_, Figure 4c), the reduction in bacterial population was further pronounced, with only scattered individual cells and occasional small rod clusters remaining visible. Importantly, a subset of the residual cells displayed irregular surface contours and less sharply defined outlines than control bacilli, suggesting morphological alterations of the bacterial cell envelope at this intermediate Nb_2_O_5_ concentration.

The most pronounced morphological effects were observed in the G3-treated group (2.0% *w*/*w* Nb_2_O_5_, Figure 4d). Bacterial density was reduced to a minimal level, with only a small number of cells detectable across the entire imaged field. The residual bacteria exhibited altered morphology, including reduced cell size and irregular surface features. These morphological changes are consistent with cellular damage following exposure to the CG-Nb hydrogels; however, SEM observations alone cannot establish membrane leakage, cytoplasmic loss, or a specific mechanism of cell death. These structural changes are consistent with the antibacterial activity observed for the CG-Nb hydrogels. However, the SEM observations do not establish the specific mechanism responsible for the observed antibacterial effects [38].

The progressive morphological changes observed from G1 to G3 were consistent with the concentration-dependent antibacterial activity observed in the colony reduction assays. The observed morphological alterations became more pronounced with increasing Nb_2_O_5_ loading, consistent with the lower colony counts observed for the CG-Nb hydrogels. The intrinsic antibacterial properties of chitosan may also contribute to the observed antibacterial activity of the composite hydrogels [39]. Collectively, the SEM observations showed concentration-dependent changes in bacterial morphology following exposure to the CG-Nb hydrogels, consistent with the quantitative antibacterial results.

Chitosan-based hydrogels are widely reported to exhibit intrinsic antibacterial activity, while incorporation of additional antimicrobial components can further enhance their antibacterial performance [40]. Previous chitosan–gelatin hydrogel systems have also demonstrated antibacterial activity and cytocompatibility against *S. aureus* and *E. coli*. In our previous work, the same CG-Nb hydrogel platform was characterized physicochemically, including structural and morphological analyses [41]. The present study extends this work by evaluating the biological performance of the platform, including antibacterial activity, photoinduced singlet oxygen generation, and cytocompatibility.

These findings are broadly consistent with previous reports showing that chitosan-based hydrogel systems can provide intrinsic antibacterial activity, while incorporation of additional functional components can enhance their antibacterial performance. In the present study, increasing Nb_2_O_5_ loading was associated with progressively lower CFU counts and larger inhibition zones compared with the unloaded CG matrix, indicating enhanced antibacterial activity under the conditions tested. The present results therefore extend the previous physicochemical characterization of the CG-Nb platform by providing a systematic biological evaluation against both Gram-positive and Gram-negative bacteria, together with assessment of photo-induced singlet oxygen generation and short-term cytocompatibility. However, direct quantitative comparison with published systems should be interpreted cautiously because differences in polymer composition, nanoparticle loading, bacterial strains, assay conditions, and exposure times can substantially influence the measured antibacterial response.

The present study was designed as an initial biological evaluation of a previously developed Nb_2_O_5_-loaded hydrogel platform. Accordingly, antibacterial activity was assessed using standardized planktonic bacterial models, while biofilm assays and multidrug-resistant clinical isolates were beyond the intended scope of the present investigation. In addition, singlet oxygen generation was evaluated using the DPA bleaching assay, which selectively detects ^1^O_2_ and does not quantify the complete spectrum of reactive oxygen species. These limitations should be considered when interpreting the current findings, which represent an initial biological evaluation of the previously developed hydrogel platform.

## 3. Materials and Methods

### 3.1. Materials

Chitosan (low-molecular-weight, ~70% deacetylated), gelatin from porcine skin (Type A), citric acid anhydrous, glutaraldehyde solution (25% in H_2_O), and 9,10-diphenylanthracene (DPA) were purchased from Sigma-Aldrich (St. Louis, MO, USA). Synthesized niobium pentoxide (Nb_2_O_5_) nanoparticles (<100 nm particle size) were used as described in the Method Section of Ref. [42]. The XTT cell viability assay kit was also sourced from Sigma-Aldrich. Phosphate-buffered saline (PBS), nutrient agar, and brain heart infusion (BHI) broth were acquired from Himedia Laboratories (Kennett Square, PA, USA). RPMI-1640 medium, fetal bovine serum (FBS), and penicillin-streptomycin (P/S) were purchased from Gibco (Waltham, MA, USA). *Escherichia coli* (ATCC 25922) and *Staphylococcus aureus* (ATCC 25923) were used as model bacteria. L929 mouse fibroblast cells (NCTC clone 929 [L cell, L-929, derivative of Strain L]; ATCC CCL-1) were obtained from the American Type Culture Collection (ATCC, Manassas, VA, USA). All chemicals were of analytical grade. Deionized (DI) water was used throughout.

### 3.2. Fabrication of Niobium-Loaded Chitosan–Gelatin (CG-Nb) Hybrid Hydrogels

The hybrid hydrogels were synthesized via a sequential dissolution, blending, and crosslinking method. A 1% (*w*/*v*) chitosan solution was prepared by dissolving 500 mg of chitosan in 50 mL of a 2% (*w*/*v*) aqueous citric acid solution with continuous stirring at 47 °C for 18–24 h. Separately, an 8% (*w*/*v*) gelatin solution was prepared by dissolving 400 mg of gelatin in 5 mL of DI water at 50 °C. The warm gelatin solution was added dropwise to the chitosan–citric acid solution under vigorous stirring to form a homogeneous chitosan–gelatin (CG) blend. A stable suspension of Nb_2_O_5_ nanoparticles (2 mg/mL) was prepared in DI water using probe sonication (200 W, 10 min, ice bath). Predetermined volumes of this suspension were incorporated into separate batches of the CG blend to create three nanocomposite formulations: G1—5 mL suspension (10 mg Nb_2_O_5_); G2—7 mL suspension (14 mg Nb_2_O_5_); and G3—9 mL suspension (18 mg Nb_2_O_5_). The plain CG hydrogel without nanoparticles served as the control. The final Nb_2_O_5_/(chitosan+gelatin) weight ratios were approximately 1.1% *w*/*w* (G1), 1.6% *w*/*w* (G2), and 2.0% *w*/*w* (G3). Glutaraldehyde was added to each mixture at 0.25% (*v*/*v*) of the total volume as a crosslinker. Glutaraldehyde was selected as the crosslinking agent because it provides efficient stabilization of chitosan–gelatin networks at a relatively low concentration. Because residual glutaraldehyde may affect biological compatibility, the formed hydrogels were thoroughly washed with deionized water after crosslinking and subsequently washed with sterile PBS before biological testing to minimize residual soluble crosslinker. After 1 h of stirring and pH adjustment to 4.9–5.0 using aqueous NaOH, the solutions were transferred to Petri dishes and allowed to crosslink for 24 h at room temperature. The formed hydrogels were washed thoroughly with DI water. For biological assays, sterile hydrogel discs (10 mm diameter, ~2 mm thickness) were prepared aseptically. The physicochemical characterization of the synthesized Nb_2_O_5_ nanoparticles, including particle size distribution, zeta potential, XRD phase analysis, and TEM morphology, as well as the structural and mechanical characterization of the resulting CG-Nb hydrogels, has been reported in detail in our previous publications [42,43] and is not repeated here. The three Nb_2_O_5_ concentrations evaluated (1.1%, 1.6%, and 2.0% *w*/*w*) were selected based on preliminary experiments and the physicochemical characterization data reported in our prior publication. Concentrations above 2.0% *w*/*w* were not evaluated because they led to nanoparticle aggregation and inhomogeneous hydrogel formation in preliminary trials.

The present study focused primarily on the biological evaluation of the previously developed Nb_2_O_5_-loaded chitosan–gelatin hydrogel platform. Therefore, additional XRD, SEM, and TGA analyses of the hydrogels before and after Nb_2_O_5_ incorporation were not performed in the present study. Similarly, new TEM imaging of the Nb_2_O_5_ nanoparticles was not conducted in this work. The relevant nanoparticle characterization, including particle-size distribution, zeta potential, XRD phase analysis, and TEM morphology, as well as structural and mechanical characterization of the CG-Nb hydrogels, has been reported in our previous publications.

#### Sterilization of Hydrogel Discs

For biological assays, sterile hydrogel discs (10 mm diameter, approximately 2 mm thickness) were prepared using a sterile cork borer under aseptic conditions. The discs were immersed in sterile distilled water for 30 min to remove residual soluble components. Following this, the discs were washed three times with sterile phosphate-buffered saline (PBS, pH 7.4) under gentle shaking (50 rpm) at room temperature. For each wash, the PBS was replaced after 10 min intervals to ensure complete removal of any remaining water-soluble contaminants. The sterilized discs were stored in sterile PBS at 4 °C until further use. Sterility was confirmed by incubating randomly selected discs on nutrient agar plates at 37 °C for 48 h; no bacterial growth was observed.

### 3.3. Antibacterial Studies

#### 3.3.1. Agar Disc Diffusion Assay

The antibacterial activity of the hydrogels was evaluated using a modified agar well diffusion method, where sterile hydrogel discs were placed directly onto the agar surface. Briefly, Mueller–Hinton agar plates were inoculated with 100 µL of a bacterial suspension (~10^6^ CFU/mL) and uniformly spread with a sterile cotton swab to create a confluent lawn. Using sterile forceps, hydrogel discs (CG, G1, G2, and G3) were gently placed onto the agar surface to ensure complete contact between each disc and the agar. The plates were incubated at 37 °C for 18–24 h. Following incubation, the diameter of the clear zone of inhibition (ZOI) around each disc was measured using a digital caliper. All experiments were performed in triplicate (*n* = 3).

#### 3.3.2. Direct Contact Colony Reduction Assay

The bactericidal efficacy of the hydrogels was quantified using a direct contact method in 6-well plates. Overnight bacterial cultures were diluted in fresh brain heart infusion (BHI) broth to achieve a final concentration of approximately 1 × 10^6^ CFU/mL. One milliliter of this bacterial suspension was added to each well of sterile 6-well plates. Sterile hydrogel discs (CG, G1, G2, G3) were placed directly into the wells using sterile forceps, ensuring complete immersion in the bacterial suspension. Control wells contained bacterial suspension without any hydrogel disc. The plates were incubated at 37 °C for 24 h. After incubation, the hydrogel discs were carefully removed. The PBS wash was performed to remove loosely suspended planktonic bacteria, while the gentle scraping step was used to recover bacteria that remained in direct contact with the well surface after hydrogel exposure, consistent with the direct-contact antibacterial assessment method. This combined recovery approach ensures quantification of residual viable bacteria present in the contact zone. A 100 µL sample was collected from each well. The samples were vortexed thoroughly, and serial 10-fold dilutions were prepared in sterile PBS up to 10^−6^. From each dilution, 100 µL was spread onto nutrient agar plates and incubated at 37 °C for 18–24 h. Viable colonies were counted, and colony-forming units per mL (CFU/mL) were calculated. The antibacterial activity was calculated as the percentage reduction in bacterial colonies using the following equation:

$$\mathrm{Reduction}\;\mathrm{rate}\;\left(\%\right)=\left(\frac{N_{c}-N_{s}}{N_{c}}\right)\times 100$$

where $N_{c}$ represents the CFU of the untreated control, and $N_{s}$ represents the CFU of the treated sample. All experiments were performed in triplicate with three independent hydrogel discs per formulation (*n* = 3).

#### 3.3.3. Evaluation of Singlet Oxygen Generation (9,10-Diphenylanthracene Bleaching Assay)

The generation of singlet oxygen (^1^O_2_) by the niobium-containing hydrogels was assessed using the 9,10-diphenylanthracene (DPA) bleaching assay. A stock solution of 200 µM DPA was prepared in absolute ethanol and stored at −20 °C in the dark. A working solution of 20 µM DPA was prepared in PBS immediately before use. For direct hydrogel assessment, sterile hydrogel discs (G3, 10 mm diameter) were placed into quartz cuvettes containing 2 mL of the 20 µM DPA working solution. PBS without any hydrogel served as the negative control, and a 10 µM methylene blue solution served as the positive photosensitizer control. The cuvettes were irradiated with a blue LED light source (450 ± 10 nm, 50 mW/cm^2^) for 30 min. Absorbance at 374 nm was measured at 5 min intervals using a UV-Vis spectrophotometer. An identical set of samples was kept in the dark for the same duration as dark controls. The percentage of DPA bleached was calculated using the formula:

$$\mathrm{DPA}\;\mathrm{bleaching}\;(\%)=\left(\frac{A_{0}-A_{t}}{A_{0}}\right)\times 100$$

where $A_{0}$ is the initial absorbance of DPA at zero time, and $A_{t}$ is the absorbance at time t after treatment, providing direct visual evidence of loss of membrane integrity and cytoplasmic leakage.

#### 3.3.4. In Vitro Cytotoxicity Assay (XTT Assay)

The cytocompatibility of the hydrogels was assessed using L929 mouse fibroblast cells in accordance with ISO 10993-5 guidelines. Cells were cultured in Dulbecco’s Modified Eagle Medium (DMEM) supplemented with 10% fetal bovine serum (FBS) and 0.5% penicillin-streptomycin (P/S) at 37 °C in a humidified atmosphere containing 5% CO_2_. Upon reaching approximately 80–90% confluence, the culture medium was removed, and the cell monolayer was gently washed with sterile PBS. Cells were detached by adding 0.25% trypsin-EDTA solution and incubating at 37 °C for 2–3 min. Detachment was confirmed under an inverted microscope. Fresh DMEM containing 10% FBS was added to neutralize the trypsin, and the cell suspension was gently pipetted to obtain a single-cell suspension. The cell number and viability were determined using a hemocytometer with trypan blue exclusion. Cells were seeded in 24-well plates at a density of 1 × 10^4^ cells per well in 1 mL of complete DMEM. The plates were incubated for 24 h at 37 °C in a 5% CO_2_ atmosphere to allow cell attachment.

After 24 h, the culture medium was aspirated from all wells. Sterile hydrogel discs (CG, G1, G2, G3, 10 mm diameter) were placed directly onto the cell monolayer using sterile forceps. Fresh DMEM (1 mL) was added gently to each well to ensure the cells remained covered. Control wells received fresh medium only without any hydrogel disc. The plates were incubated for an additional 24 h at 37 °C in a 5% CO_2_ incubator. Following the 24 h incubation, the hydrogel discs were carefully removed from each well. The XTT labeling mixture was prepared fresh according to the manufacturer’s instructions by mixing the XTT labeling reagent with the electron-coupling reagent in a 50:1 ratio. An appropriate volume of the XTT reaction mixture was added to each well. The plates were incubated for 1.5 h at 37 °C in the dark. After incubation, the plates were gently shaken, and aliquots were transferred to a 96-well plate for absorbance measurement at 450 nm with a reference wavelength of 630 nm using a microplate reader. Cell viability was determined by measuring the absorbance of treated and control wells using a microplate reader at the specified wavelength. The direct contact method was selected because it represents a stringent worst-case exposure condition, appropriate for hydrogel biomaterials designed for direct tissue-contact applications. This method is explicitly sanctioned under ISO 10993-5 and has been widely used for the cytocompatibility evaluation of hydrogel-based biomaterials [44].

Glutaraldehyde was selected as the crosslinking agent because its aldehyde groups can react with amino groups present in chitosan and gelatin, promoting stabilization of the polymeric network at a relatively low concentration. Although the hydrogels were extensively washed with deionized water and sterile PBS before biological testing, residual glutaraldehyde was not directly quantified in the present study. Therefore, the possibility that residual crosslinker contributed to the observed biological response cannot be completely excluded.

The percentage of viable cells was calculated relative to the untreated control group according to the following equation:

$$\mathrm{Cell}\;\mathrm{viability}\;(\%)=\left(\frac{A_{\mathrm{sample}}}{A_{\mathrm{control}}}\right)\times 100$$

where $A_{\mathrm{sample}}$ represents the absorbance of cells treated with the hydrogel extract and $A_{\mathrm{control}}$ represents the absorbance of untreated control cells.

### 3.4. Scanning Electron Microscope Analysis

The surface morphology of bacterial cells following exposure to Nb_2_O_5_ nanoparticle hydrogels was evaluated using a Helios Nano Lab 650 field-emission scanning electron microscope (FEI, Hillsboro, OR, USA). Bacterial samples were prepared by applying the test hydrogels (experimental groups) and hydrogel-free medium (control groups) to sterile coverslips, followed by incubation under appropriate growth conditions. After the designated exposure period, samples were fixed with 2.5% glutaraldehyde for 2 h at 4 °C, followed by post-fixation with 1% osmium tetroxide for 1 h. Dehydration was performed through a graded ethanol series (30%, 50%, 70%, 90%, 95%, and 100% *v*/*v*), followed by a final dehydration step with 100% isopropanol. Dried samples were mounted on aluminum stubs using double-sided carbon tape and sputter-coated with a 5 nm layer of gold-palladium alloy to enhance conductivity and prevent charging. Imaging was performed using the instrument’s Schottky field-emission electron source, operated at 3 kV in high-resolution mode, with a working distance of 2.8 mm to optimize surface detail while minimizing charging artifacts. The instrument, which also features a gallium-focused ion beam (FIB) column and an Oxford Instruments X-Max energy-dispersive X-ray spectroscopy (EDS) detector for elemental analysis, provides a resolution of 0.8–1.5 nm across operating ranges of 30 kV to 200 V.

### 3.5. Statistical Analysis

All experiments were performed in triplicate, and results were expressed as mean ± standard deviation (SD). Statistical analysis was performed using one-way analysis of variance (ANOVA), followed by Tukey’s post hoc test for multiple comparisons, in GraphPad Prism version 9.0 (GraphPad Software, Boston, MA, USA). Differences were considered statistically significant at *p* < 0.05. The coefficient of variation (CV) was calculated as (SD/mean) × 100% to assess experimental reproducibility.

## 4. Conclusions

This study evaluated the antibacterial activity, photo-induced singlet oxygen generation, and in vitro cytocompatibility of previously developed niobium pentoxide-loaded chitosan–gelatin (CG-Nb) hydrogels. Incorporation of Nb_2_O_5_ was associated with progressively greater antibacterial activity across the evaluated concentration range against both *Staphylococcus aureus* and *Escherichia coli*, as demonstrated by agar diffusion, direct-contact colony reduction, and SEM analyses.

Among the evaluated formulations, G3 (2.0% *w*/*w* Nb_2_O_5_) showed the highest antibacterial activity and maintained 87.3 ± 4.8% L929 fibroblast viability after 24 h of exposure, remaining above the ISO 10993-5 cytotoxicity threshold under the tested conditions. The DPA bleaching assay demonstrated that G3 can generate photo-induced singlet oxygen under blue-light irradiation. However, because the antibacterial assays were conducted without external irradiation, the present findings do not establish a direct contribution of photo-induced singlet oxygen to the observed antibacterial activity. The SEM observations revealed morphological changes in bacteria following hydrogel exposure that were consistent with the quantitative antibacterial results, but they do not establish a specific mechanism of bacterial damage.

The present study provides an initial biological evaluation of the previously developed Nb_2_O_5_-loaded chitosan–gelatin hydrogel platform. The findings demonstrate antibacterial activity and short-term cytocompatibility under the experimental conditions investigated. However, the study was limited to planktonic bacterial models, a 24 h XTT cytocompatibility assessment, and singlet oxygen evaluation of the G3 formulation under blue-light irradiation. Further studies involving matched light and dark antibacterial conditions, longer-term cytocompatibility, biofilm models, and mechanistic investigations would be required to establish the broader biological performance and potential biomedical applications of these hydrogels.

## Figures and Tables

**Table 1:** Direct Contact Colony Reduction Assay Results for Hydrogel Formulations.

Formulation	Nb_2_O_5_ (% *w*/*w*)	*S. aureus* CFU Count	Reduction (%)	*E. coli* CFU Count	Reduction (%)
**Control**	0	>300	-	>300	-
**CG**	0	>300	ND †	>300	ND †
**G1**	1.1	53 ± 8.5 ^a^	82.3 ± 2.8 ^a^	56 ± 9.2 ^a^	81.3 ± 3.1 ^a^
**G2**	1.6	37 ± 6.2 ^b^	87.7 ± 2.1 ^b^	32 ± 5.5 ^b^	89.3 ± 1.8 ^b^
**G3**	2.0	13 ± 3.1 ^c^	95.7 ± 1.0 ^c^	20 ± 4.2 ^c^	93.3 ± 1.4 ^c^

Values represent mean ± standard deviation (*n* = 3). Control and CG counts exceeded 300 CFU (too numerous to count, TNTC). Reduction percentages for G1–G3 were calculated using the minimum detectable threshold (300 CFU) as Nc, providing conservative estimates of bactericidal activity. Different superscript letters within the same column indicate statistically significant differences (*p* < 0.05) as determined by one-way ANOVA with Tukey’s post hoc test. CG colonies were too numerous to count precisely (>300 CFU); therefore, an exact CFU reduction could not be calculated for the CG formulation under the direct-contact assay conditions. † ND, not detected.

**Table 2:** XTT assay absorbance readings and cell viability for control and hydrogel-treated groups.

Group	Mean Absorbance (OD) ± SD	Cell Viability (%) ± SD
**Control**	3.425 ± 0.235	100.0 ± 6.9
**G1**	3.376 ± 0.120	98.6 ± 3.5
**G2**	3.226 ± 0.140	94.2 ± 4.1
**G3**	2.990 ± 0.164	87.3 ± 4.8

## Data Availability

The data presented in this study are available from the corresponding author upon reasonable request.

## Supplementary Materials

The following supporting information can be downloaded at https://www.mdpi.com/article/10.3390/ijms27209124/s1.

## Abbreviations

ANOVA, analysis of variance; ATCC, American Type Culture Collection; BHI, brain heart infusion; CFU, colony-forming units; CG, chitosan–gelatin; CG-Nb, niobium pentoxide-loaded chitosan–gelatin; CV, coefficient of variation; DI, deionized; DMEM, Dulbecco’s Modified Eagle Medium; DPA, 9,10-diphenylanthracene; *E. coli*, *Escherichia coli*; EDS, energy-dispersive X-ray spectroscopy; FE-SEM, field-emission scanning electron microscopy; FBS, fetal bovine serum; GA, glutaraldehyde; G1, hydrogel containing 1.1% *w*/*w* Nb_2_O_5_; G2, hydrogel containing 1.6% *w*/*w* Nb_2_O_5_; G3, hydrogel containing 2.0% *w*/*w* Nb_2_O_5_; ISO, International Organization for Standardization; L929, mouse fibroblast cell line; LED, light-emitting diode; LPS, lipopolysaccharide; Nb_2_O_5_, niobium pentoxide; OD, optical density; PBS, phosphate-buffered saline; P/S, penicillin–streptomycin; rpm, revolutions per minute; *S. aureus*, *Staphylococcus aureus*; SD, standard deviation; SEM, scanning electron microscopy; TNTC, too numerous to count; UV–Vis, ultraviolet–visible spectroscopy; XTT, 2,3-bis(2-methoxy-4-nitro-5-sulfophenyl)-2H-tetrazolium-5-carboxanilide; ZOI, zone of inhibition; ^1^O_2_, singlet oxygen.
